# Supporting general practitioner-based care for poorly controlled type 2 diabetes mellitus (the DECIDE study): feasibility study and protocol for a pilot cluster randomised controlled trial

**DOI:** 10.1186/s40814-018-0352-y

**Published:** 2018-10-13

**Authors:** Mark E Murphy, Molly Byrne, Fiona Boland, Derek Corrigan, Paddy Gillespie, Tom Fahey, Susan M Smith

**Affiliations:** 1 HRB Centre for Primary Care Research, Royal College of Surgeons, Dublin, Ireland; 2 Health Behaviour Change Research Group, School of Psychology, National University of Ireland, Galway, Ireland; 3 Health Economics and Policy Analysis Centre (HEPAC), National University of Ireland, Galway, Ireland

## Abstract

**Background:**

Poorly controlled type 2 diabetes mellitus (T2DM) is associated with significant morbidity, mortality and healthcare costs. Control of T2DM can be challenging for healthcare professionals for a number of reasons, including poor concordance with medications, difficulties modifying lifestyle behaviour and also clinical inertia, which is defined as a reluctance among health professionals to intensify medications. A complex intervention, called ComputeriseD dECisIonal support for poorly controlleD typE 2 Diabetes mellitus in Irish General Practice (DECIDE), was developed, identifying T2DM patients with poor glycaemic and blood pressure control and aiming to target clinical inertia, by supporting therapeutic action, including GP-led medication intensification where appropriate. A small-scale, uncontrolled, non-randomised feasibility study highlighted the acceptability of the DECIDE intervention within Irish General Practice. This paper presents a protocol for a pilot cluster randomised controlled trial (RCT) of the DECIDE intervention.

**Methods/Design:**

The pilot cluster RCT will involve 14 practices and 140 patients in Irish General Practice. Intervention GPs will participate in the DECIDE intervention, comprising (a) a training programme for the practices and (b) a web-based clinical decision support system supporting treatment escalation, tailored to specific patient information. Only patients who have poorly controlled T2DM (defined as HbA1c > 70 mmol/mol and/or BP > 150/95) will be included. The primary outcomes will include measures of feasibility such as recruitment and retention of practices and acceptability of the intervention and also HbA1c. Secondary outcomes will include medication intensification, blood pressure and lipids. Control GPs will continue to provide usual care. A process evaluation will be performed to determine whether the intervention is delivered as intended and treatment fidelity assessed to monitor and enhance the reliability and validity of interventions. An exploratory health economic analysis will examine the potential costs and cost effectiveness of the intervention relative to the control.

**Discussion:**

A pilot cluster RCT will establish the feasibility of a complex intervention which aims to support primary care for patients with poorly controlled T2DM in Irish General Practice.

**Trial registration:**

The protocol for the pilot cluster RCT is registered on the ISRCTN Registry at: ISRCTN69498919.

## Background

Over 3.7 million deaths globally were attributable to diabetes in 2012; two thirds of these deaths were caused by the micro- and macro-vascular complications of poorly controlled diabetes [1]. Despite clear evidence to support the management of type 2 diabetes mellitus (T2DM) to prevent its complications, many patients continue to have poor control of glycaemic and cardiovascular risk factors (including blood pressure (BP) and lipids), which are strong surrogate measures of morbidity, mortality, poor quality of life and increased economic burden for patients and health systems [1–4]. Some studies indicate that substantial proportions of patients with T2DM continue to have poor glycaemic and blood pressure control for several years before intensification with therapeutic agents occurs [2, 4, 5].

The reasons for poor control of T2DM are multiple, including a reluctance to intensify medications by a physician, poor adherence and concordance with medications and difficulties modifying lifestyle behaviours [6]. Clinical inertia is one cause of poor control of T2DM and is defined as a failure to apply evidence-based guidance and intensify treatment, and it is an impediment to efficient care [7–9]. The need to intensify therapies in an appropriate and timely manner is a prerequisite to improving the management of T2DM [10–15]. Significant delays in the intensification of oral hypoglycaemic, anti-hypertensive and lipid-lowering medications have been demonstrated [2, 16–20]. The availability of multiple new medications has created a difficulty for physicians, in terms of choice and the complexity of decision-making, with many patients requiring two to three anti-glycaemic medications [3, 21]. A recent network meta-analysis has shown that newer agents such as GLP-1 agonists and SGLT-2 inhibitors are associated with improved mortality [22]. There is also emerging evidence of geographical variations in prescribing anti-diabetic medication, indicating a degree of variation in effective care [23].

### Rationale for the DECIDE study

Assessing glycaemic control over the preceding months, with a HbA1c test, is recommended in all T2DM guidelines, with a lower HbA1c (e.g. below 58 mmol/mol) [3]. Intensive control of glycaemic and cardiac risk factors can reduce mortality, but aggressive reductions in HbA1c, for all patients, may achieve more harm than benefit in certain patient populations [24]. Therefore, targeted reductions in cardiovascular and glycaemic risk factors in certain vulnerable populations (e.g. cognitively impaired) and those with very poorly controlled T2DM have been advocated [25, 26]. A systematic review was performed which sought to understand the effect of healthcare interventions specifically targeting patients with poorly controlled T2DM in primary care and included 38 randomised control trials (RCTs) [26]. It showed that healthcare interventions targeting patients with poorly controlled T2DM have positive, albeit modest, effects on HbA1c with a mean difference (MD) in HbA1c − 3.7 mmol/mol, favouring healthcare interventions over usual care. Interventions targeting those with a higher baseline HbA1c (over 80 mmol/mol) had more beneficial effects (MD − 6.3 mmol/mol). The review suggested that organisational-predominant interventions were more effective than patient-centred interventions, despite patient-related factors being associated with poor control of T2DM. There was only one professional-based intervention, which studied the effect of a patient decision aid [27].

Clinical decision support systems (CDSSs) involve computer software designed to support decision-making, matching individual patient characteristics to a computerised clinical knowledge base and then providing patient-specific assessments or recommendations to the clinician to support a decision that can relate to diagnosis, investigation, prognosis or treatment [28]. Two systematic reviews have previously examined the impact of CDSS on the management of T2DM in primary care—between them looking at 28 trials [29, 30]. They highlighted multi-faceted CDSS interventions with varying results. The first review contained 15 studies, between 2010 and 2012. It found small effects with low quality of evidence. It concluded that there was a need for ‘more potent methods of decision support’ [30]. The different CDSS interventions were too heterogeneous to pool. Only one study by O’Connor et al. involved a CDSS intervention designed to target evidence-based prescribing for general practitioners, but did not focus on those with poor control and also involved practice nurses [30, 31]. The second review contained 20 studies from 1990 to 2011. Processes of case were found to improve in low-risk populations, with the use of CDSS in diabetes management, without clear evidence of improving patient outcomes [29]. None of the CDSS interventions were designed to promote intensification of prescribing in persons with poor glycaemic control [32].

### Development of the DECIDE study and non-randomised feasibility study

We developed a theory-based complex intervention, called DECIDE: ComputeriseD dECisIonal support for poorly controlleD typE 2 Diabetes mellitus in Irish General Practice. The first stage of the UK Medical Research Council (MRC) framework was used to develop the intervention, using the Behaviour Change Wheel (BCW) as a theoretical guide to intervention development [33–35]. The intervention development is reported elsewhere and incorporates a CDSS element, which is professionally targeted at general practitioner (GP) prescribing behaviour, in the treatment of patients with poorly controlled T2DM [36].

We conducted a small-scale, uncontrolled, non-randomised feasibility study to assess the feasibility and acceptability of the DECIDE intervention within Irish General Practice [36]. Table 1 summarises the provision of T2DM healthcare in Ireland. Four GPs, including three academic GPs and one full-time clinical GP, piloted the DECIDE intervention. Each GP applied the intervention process to 15 different patients with poor control of T2DM. All practices were affiliated with the Health Research Board (HRB) Centre for Primary Care Research (http://www.hrbcentreprimarycare.ie). We included all patients who had both HbA1c > 70 mmol/mol and systolic blood pressure (SBP) > 150 mmHg or diastolic blood pressure (DBP) > 95 mmHg. Whilst there is no validated definition of poor control in the literature, we defined it as a HbA1c reading of ≥ 70 mmol/mol and a BP level of ≥ 150/95 mmHg [36]. When this number of patients was < 15, we included a stratified randomised sample of patients with only HbA1c > 70 mmol/mol or SBP > 150 mmHg or DBP > 95 mmHg. The list of 15 patients was kept anonymously within the practice.

**Table 1:** Access to healthcare and structure of T2DM care in the Republic of Ireland

Access to general practice healthcare in the Republic of Ireland	
• The General Medical Services (GMS) scheme provides medical care to approximately 40% of the Irish population. It is predominantly means-tested and provides those who are eligible with free general practitioner visits, free hospital care and free medications (except for a prescription levy, currently €2.50 per item to a maximum of €25). A further ~ 5% of the population are entitled to free doctor visits (called a Doctor Visit Card (DVC)) based upon means testing and age-banding (all under-6-year-olds and over-70-year-olds). • The Long Term Illness (LTI) scheme allows persons with certain medical conditions (T2DM being one) to have free access to medications which treat that condition. All patients with type 2 diabetes mellitus (T2DM) can have free medications under the LTI scheme. • The GMS and LTI schemes are administered by the Health Services Executive (HSE) and Primary Care Reimbursement Services (PCRS). • ‘Private patients’ represent approximately 45% of the population and are not entitled to a GMS or DVC card, paying the full cost for attending a GP, out-of-pocket, at the point of healthcare delivery.	
Structure of diabetes care in Republic of Ireland	
• Before October 2015, structured chronic disease management of T2DM was not universally available in Irish primary care. Approximately ten primary care schemes existed in 2013 and 2014, providing different levels of structured T2DM care, often set up as pilot schemes. This represented a maximum of 250 practices within Irish general practice (approximately 10% of total practices). Up until October 2015, the vast majority of structured T2DM care in Ireland was provided in secondary care, through public hospital outpatients or under the care of endocrinologists in private clinics. • In October 2015, a new agreement was reached with GPs entitling all GMS patients to a structured diabetes programme in primary care (called a Diabetes Cycle of Care) with two free GP visits per annum. Private patients with T2DM either pay to receive care from their GP or continue to attend secondary care.	

The non-randomised feasibility study included 15 patients with poor control of T2DM per practice; the sample was generated through a random stratified number of the eligible patients with poor control of either glycaemic and/or BP risk factors. This was performed remotely with no identifiable patient information leaving the practice. The pilot GPs contacted the study team (MM and FB) with the number of persons included in the finder tool report and were informed which 15 patient IDs to include in the pilot study. The finder tool software was developed through a collaboration with the electronic health record (EHR)-vendors and the study team. Each practice was issued with a secure, specific login for the DECIDE website, which housed the CDSS. Approximately one clinical session was spent by pilot GPs performing the DECIDE intervention for all the included 15 patients. Firstly, GPs inserted clinical information for each patient into the DECIDE website, including anonymous baseline information and clinical information necessary for the CDSS (e.g. HbA1c, BP and medications). The second step for each patient involved analysing the suggested treatment intensification options from the CDSS on the DECIDE website. The options were based upon the Irish College of General Practitioners (ICGP) and National Institute for Health and Clinical Excellence (NICE) guidelines, linking the patient’s clinical information and medications to the evidence base for anti-glycaemic, anti-hypertensive and lipid-lowering medications [36]. Choosing an intensification option or acting on a recommendation was at the discretion of each GP.

A total of 37 patients from four practices with ‘poorly controlled’ T2DM received the DECIDE intervention and had complete data for analysis in the practice pilot study. Table 2 summarises the baseline patient’s demographics, highlighting the opportunities to intensify medications. The mean HbA1c was 83.9 mmol/mol, but most patients had mild hypertension (mean 141/83 mmHg) and a normal total cholesterol (mean 4.7 mmol/L). A substantial number of patients were either on no medications or monotherapy for glycaemic and BP management.

**Table 2:** Summary of baseline characteristics and medications in the non-randomised feasibility study

Age	56.7 years (13.7) Mean (SD)		
Gender	Male (67%)		
Duration diabetes	7.1 years (5.3) Mean (SD)		
GMS Status	69.2%		
HbA1c level	83.9 mmol/mol (30.8) Mean (SD)		
SBP	140.9 mmHg (22.71) Mean (SD)		
DBP	83.4 mmHg (20.5) Mean (SD)		
Total cholesterol	4.7 mmol/L (1.8) Mean (SD)		
Prevention status	Primary prevention (81%)		
Glycaemic medications (37 patients valid)	No medications	8.1% (*n* = 3)	–
	Monotherapy (without insulin)	32.4% (*n* = 12)	Metformin (*n* = 2)
	Dual therapy (without insulin)	21.6% (*n* = 8)	Metformin + sulphonylurea (*n* = 5) Metformin + DPP-4 inhibitor (*n* = 2) Sulphonylurea + DPP-4 inhibitor (*n* = 1)
	Triple therapy (without insulin)	8.1% (*n* = 3)	Metformin + sulphonylurea + DPP-4 inhibitor (*n* = 3)
	Quadruple therapy (without insulin)	2.7% (*n* = 1)	Metformin + sulphonylurea + DPP-4 inhibitor + SGLT2 inhibitor (*n* = 3)
	Insulin ± other medication(s)	27.0% (*n* = 10)	
Anti-hypertensive medications (37 valid patients)	No medications	24.3% (*n* = 9)	
	Monotherapy	35.1% (*n* = 13)	ACE-inhibitor* (*n* = 11) Beta-blocker (*n* = 2)
	Dual therapy	13.5% (*n* = 5)	ACE-inhibitor + diuretic (*n* = 2) ACE-inhibitor + CCB (*n* = 2) Beta-blocker + CCB (*n* = 1)
	Triple therapy	24.3% (*n* = 9)	ACE-inhibitor + CCB + beta-blocker (*n* = 4) ACE-inhibitor + diuretic + beta-blocker (*n* = 2) ACE-inhibitor + CCB + diuretic (*n* = 1) ACE-inhibitor + CCB + alpha-blocker (*n* = 1) CCB + beta-blocker + ‘Other’ anti-hypertensive (*n* = 1)
	Quadruple therapy	2.7% (*n* = 1)	ACE-inhibitor + CCB + diuretic + diuretic (*n* = 1)
Lipid-lowering medications (37 valid patients)	No medication	27% (*n* = 10)	
	Monotherapy	70.3% (*n* = 26)	Statin (*n* = 23) Ezetamibe (*n* = 2) Fibrate (*n* = 1)
	Dual therapy	2.7% (*n* = 1)	Statin + fibrate (*n* = 1)

Feedback from GPs, through interviews and structured written feedback, reported successful patient identification facilitated by the finder tool. One pilot GP stated the ‘finder tool was excellent and the decisional support was educational’. Feedback indicated that the divisions of intensification options in terms of glycaemic, blood pressure and lipid-lowering medications were clear and useful. All GPs thought that the initial step, of inserting information from the EHR, could be enabled by the practice nurse, allowing the GP to review this information and to assess the treatment intensification options from the CDSS in the second step. For some patients, it was noted the biggest issue was not a pharmacological, clinical or intensification issue; the GPs reported that the reasons for poor control related to ‘logistics’ and ‘an educational and compliance deficit which is not easily fixed with a structured six-month review’. Using DECIDE was also felt to be very quick, which was positive.

We used the results of the non-randomised feasibility study to modify processes in the next phase of the DECIDE intervention (the pilot cluster RCT (cRCT)). Therapeutic intensification actions were deemed not possible in approximately one third of patients, due to complex social reasons. Other strategies or actions were suggested, including referral to a community-based diabetes nurse specialist, asking for more frequent reviews and contacts with the patient and addressing addiction or frailty issues. These non-pharmacological ‘actions’ were deemed important to explore and document, prior to considering therapeutic intensification options. The non-randomised feasibility study raised the issue of whether the study should include patients with elevated BP, but with controlled HBA1c, as was done in the non-randomised feasibility study. Based on the pilot results, we decided to target only patients with HbA1c > 70 mmol/mol, as only a small number of patients had both raised BP and HbA1c and including patients with very elevated BP would increase our sample size requirement significantly for the pilot cRCT. We also identified additional data to be collected at follow-up, including consultation visitation rate and frequency of review at hospital and with community diabetes nurse specialists. Table 3 outlines the major changes made to the intervention based upon the results of the non-randomised feasibility study.

**Table 3:** Summary of DECIDE intervention changes

Pilot component	Issue identified in non-randomised feasibility study	Change for future pilot cluster randomised controlled trial
DECIDE finder tool	Extension of utilisation to other electronic health records (EHRs) (beyond the Socrates EHR)	A bespoke Finder Function to enable practices utilising other EHRs was developed.
DECIDE CDSS and treatment escalations options	The three domains of suggested intensification options (glycaemic, anti-hypertensive and lipid-lowering medications) were appropriate, and the CDSS was deemed useful to prompt GPs on what evidence-based intensification options were available.	Not applicable.
Non-pharmacological options	Therapeutic intensification actions were deemed not possible in approximately one third of patients, due to complex social reasons.	The DECIDE intervention actions were comprised of three intensification options for glycaemic, BP and lipid-lowering medications. A forth option—providing options of non-pharmacological actions—was added to the intervention. Examples of non-pharmacological actions included referral to a community-based diabetes nurse specialist, asking for more frequent reviews and contacts with the patient or calling the patient in for another review to discuss compliance.
Follow-up of patients	Some patients with poor control were found to have significant care needs, which would require more frequent review.	An increase in the frequency of structured visits for these persons, through individualised reviews, was recommended. Though this is a contractual matter for the GPs concerned in terms of the provision of diabetes care, the DECIDE intervention was modified to enable multiple reviews—not just one review every 6 months.
Introductory educational information on the DECIDE website, in the educational videos and DECIDE practice folder	The educational information in the DECIDE folder was deemed useful.	Some minor additions to this folder, to include the above information, were added.

## Methods/Design

### Objectives of study

The primary aim of the pilot cRCT is to test the feasibility of a complex intervention that will identify T2DM patients in Irish primary care who have poor glycaemic control and support general practitioner (GP) treatment escalation where appropriate, targeting clinical inertia in physicians. The protocol outlines the pilot cRCT, which incorporates a planned process evaluation that will record GP decision-making processes and a description of an exploratory health economic analysis that will be conducted alongside the pilot cRCT. The pilot cRCT will also inform the conduct and sample size of a definitive trial using formal continuation criteria [37].

### Study design

The next phase of the DECIDE study will be conducted through a two-arm pilot cRCT, incorporating a process evaluation and exploratory economic analysis. A cluster design will be used to reduce contamination between the arms of the trial as the intervention targets GPs. It will be conducted using the CONSORT statement, extended for use in cluster RCTs [37]. The study has been registered at http://www.isrctn.com/ISRCTN69498919.

### Setting

The intervention will take place in Irish General Practice, including practices affiliated with the HRB Primary Care Clinical Trials Network Ireland (CTNI) (http://primarycaretrials.ie), in practices using one of two EHRs (Socrates and Health One) across Ireland. We will include both single-handed GPs and group practices.

### Population

GPs will be the target of the intervention and will be the unit of randomisation (the clusters).

Patients with poorly controlled T2DM will be targeted by the GP with the DECIDE intervention. All included patients must have a diagnosis of T2DM, identified by the finder tools in the EHR (discussed above). They must be aged over 18 years and less than 75, as there is less evidence about treatment intensification in patients aged above 75 years. However, patients with both a HbA1c > 70 mmol/mol and a BP > 150/ 95 mmHg will be recruited initially to the study, followed by those with an elevated HbA1c if the total number of patients with both risk factors poorly controlled is less than ten, followed by those with just elevated BP. Not all patients will be suitable for treatment intensification, and individual decisions on treatment changes will be made and recorded by each patient’s GP.

### Randomisation

**Fig. 1:**
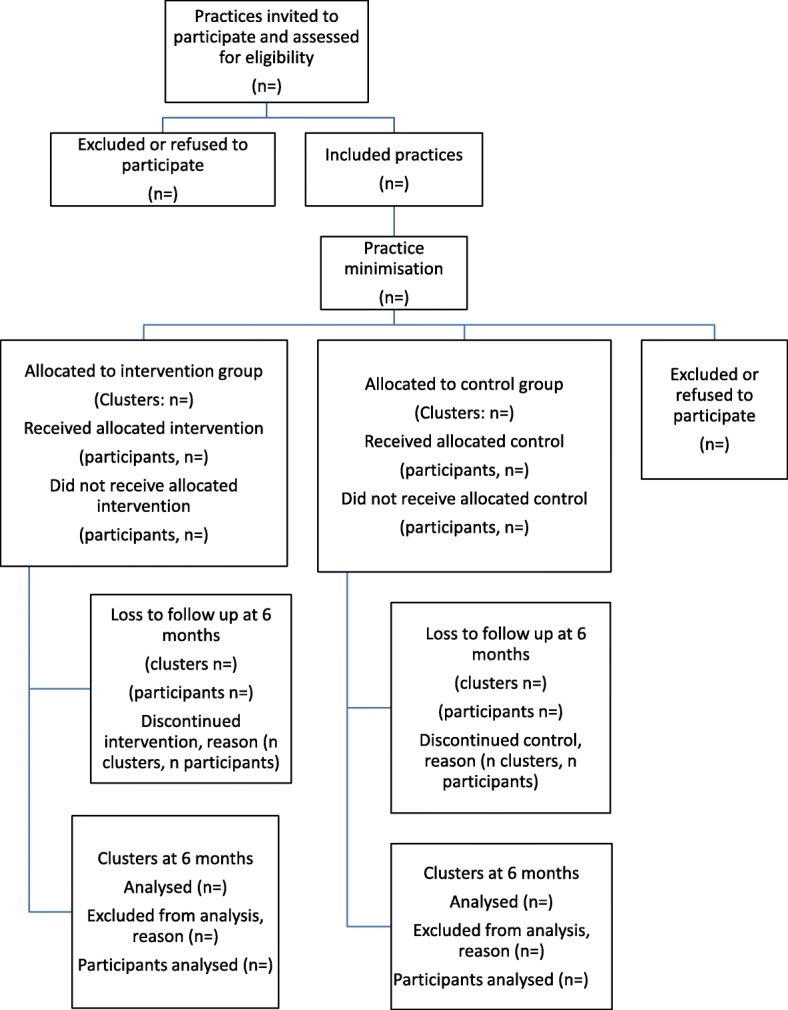
Flow of practices through the DECIDE randomised pilot study

Due to the small number of clusters, participating GPs will be allocated to the intervention or control group using minimisation (incorporating a random element). Practices will be minimised based on practice size (small/large) and involvement with structured diabetes care (see Fig. 1). Baseline data collection of the cluster units (practices) will take place before allocation. Sequence generation and practice allocation will be carried out remotely by a statistician independent of the trial management team.

### Intervention

DECIDE is a complex intervention, which used the Medical Research Council framework for development of a complex intervention, using a theoretical base and incorporating the results of simulation and a practice pilot [36]. The intervention development phase was built upon the results of the systematic review, which showed that there was only one professionally targeted intervention for patients with poor control of T2DM and none using a CDSS [26]. All practices (control and intervention) will use the finder tool to identify the included patients in the study. Table 4 outlines the intervention components in the pilot cRCT. All medication changes, which are reflected in existing guidelines, will be at the discretion of the intervention GPs.

**Table 4:** Components of the DECIDE intervention

a) A web-based decision CDSS was created which delivers patient-specific recommendations to the GP on what medication intensifications could be recommended, if appropriate. The algorithms in the CDSS are based on NICE guidance for management of hypertension and T2DM. The changes to the DECIDE intervention based upon the practice pilot study are outlined in Fig. 1. b) A training module was developed for intervention group GPs to explain all the steps in the intervention. Web-based training information and videos will be available on the DECIDE website, which houses the CDSS. An educational folder will be delivered to each GP on how to operate the CDSS.	

### Control

Control practices will continue to provide diabetes care as usual in their General Practice. They will have telephone contact from the research team to support baseline data collection. Their next contact will be at follow-up data collection, which will be at the same time as for intervention practices. Both control and intervention practices will receive a research stipend, to reflect the workload involved in the trial.

### Outcome measures

As this is a pilot study, we will ensure the feasibility and acceptability of the intervention with the GPs. The primary outcomes will be measures of feasibility such as recruitment and retention of practices and acceptability of the intervention to the GPs and also HbA1c, a measure of glycaemic control in the patient. Secondary outcomes will include changes in glycaemic medications, blood pressure medications, lipid-lowering medications and healthcare utilisation.

### Data collection

GPs and practice nurses will collect baseline data prior to allocation. Intervention GPs will then initiate the DECIDE pathway, over a 1-month window, as part of the T2DM Cycle of Care [36]. Anonymised demographics and patient information, such as patient gender and morbidity will be collected in the DECIDE website, by the practice nurse. No identifiable patient information will be made available to the researchers and will be collected for each patient at an individual level. Outcomes will be measured at 3-month follow-up, following the end of the initial 1-month intervention window.

### Sample size

As this is a pilot cRCT, a formal sample size calculation is not officially required [37]. However, based on the non-randomised feasibility study, we have calculated a provisional sample size required to estimate a clinically significant change in HBa1c. Participants in the non-randomised feasibility study who had poorly controlled T2DM had a mean baseline HbA1c of 91.1 mmol/mol (SD 16.7). Considering a clinically meaningful reduction of 10 mmol/mol in HbA1c, an intra-cluster coefficient (ICC) of 0.027 (based on a previous study [38]), using a power of 80% and allowing for a potential 10% loss to follow-up, we would need 14 practices with 10 patients in each practice giving a total of 140 patients.

### Trial process

The intervention will take place over 1 month following allocation. Follow-up data collection will take place 4 months after the intervention finishes in the intervention practices and contemporaneously in control practices, by participating practice staff, including practice nurses.

### Blinding

Care providers cannot be blinded due to the nature of the intervention but contamination of patients will be prevented using a cluster design. Patients will be aware that their practice is participating in the study but as no individually identifiable patient data is being used, individual patient consent is not being sought. Blinding of outcome assessors will not be required for the primary outcome as it is collected using an automated objective laboratory measure (HbA1c).

### Data analysis

Practice and patient-chart information will be collected. Baseline data from the practice (cluster units) will be collected prior to allocation using a practice-based questionnaire. Patient-level outcomes will be collected after allocation, using the DECIDE website, inputted by the practice nurse, then general practitioner and anonymised at practice level. Due to the nature of the project and targeting of the intervention at cluster level, patient-reported outcome measures will not be collected, as this would be beyond the scope of the proposed project and would require individual patient consent.

All results will be collected at practice level by uploading anonymised patient data through the web-based DECIDE system. Patient data is recorded at the individual level. Initially, appropriate descriptive statistics (for example, means (SD), medians (IQR) and frequencies and percentages) will be used to assess balance between the intervention and control for both practice and patient characteristics. Descriptive statistics will also be used to assess measures of feasibility such as recruitment and retention of practices.

Mixed effects regression models will be used to account for the hierarchical nature of the data. The primary analysis is to estimate HbA1c for the intervention group versus the control, adjusting for baseline HbA1c and minimisation factors. A random effects linear regression will be used, including a random practice effect to account for the correlation between patients in practices. In a secondary analysis, we will further adjust for any variables displaying an imbalance between groups at baseline. Both intention to treat (i.e. including all randomised practices and patients, regardless of participation in the intervention) and per protocol analysis (to explore whether adherence to the intervention influences the effect of the intervention) will be conducted.

The above analyses will be repeated for secondary outcomes (BP, lipids), healthcare utilisation (including practice and hospital visitation rates) and medication intensification. All analyses will use appropriate (that is, logistic or linear) regression models, with results presented as point estimates (odds ratios or difference in means), 95% confidence intervals and *p* values. The primary analyses will involve intention-to-treat comparisons between the two groups, and a secondary per protocol analysis will also be conducted [39].

Furthermore, we will conduct subgroup analyses investigating whether there is any differential effect of the intervention based on gender and age (< 65 and ≥ 65). Data analysis will be conducted once all follow-up is complete. There are no planned interim analyses. Stata v14 will be used for all analyses [40].

### Process evaluation

A process evaluation will be undertaken to determine whether the intervention is delivered as intended. Treatment fidelity is the strategy used to monitor and enhance the reliability and validity of interventions. This evaluation will address important questions regarding GP decision-making on treatment escalation. It may not always be appropriate to escalate treatment in the context of multimorbidity and other patient-related factors, and data on decision-making will be collected, whether treatment escalation occurs or not.

The process evaluation will include qualitative analysis and quantitative measures of treatment fidelity, collected through the website activity as part of the DECIDE treatment algorithms [41]. Qualitative methods will be used to explore the GP’s experience of participating in and delivering the DECIDE intervention. Semi-structured interviews (by MM) will be conducted with each participating GP. The collection of data will take place during the intervention and at 2 months after intervention completion, interviewing GPs and practice nurses from participating practices. Interviews will be conducted using telephone or in person and will be audio recorded. The topic guide will include the context to the intervention, fidelity and implementation and participant experiences. A thematic analysis will be conducted using normalisation process theory, to understand how the intervention was embedded in routine clinical practice [42]. NVivo will be used to organise and index the data.

### Health economic analysis

A preliminary health economic analysis will be conducted to compare the alternative treatment strategies: (1) *intervention*, DECIDE intervention plus usual GP care, and (2) *control*, versus usual GP care alone. The evaluation will identify, measure, value and compare the costs and outcomes of the alternatives being considered. A healthcare perspective will be adopted with respect to costing with a range of health service contacts recorded and valued, including GP use, hospital admissions, attendances at outpatient clinics and emergency clinics, and drug prescriptions. In addition, a micro-costing process will be undertaken to capture resource use relating to the operation and delivery of the DECIDE intervention. Unit costs will be applied to convert data on resource use to resource costs, and total cost variables will be calculated for both arms. For the exploratory analysis of cost effectiveness, data on total costs and the primary outcome measure of the HbA1c will be employed in an incremental analysis to explore the mean differentials in costs and effects between the intervention and the control. Univariate and multivariate sensitivity analyses, in addition to cost effectiveness acceptability curves, will be employed to address the uncertainty in the study. The output from the pilot study will provide information on the potential costs and cost effectiveness of the intervention, as well as the feasibility and acceptability of the proposed health economic approach and analysis plan, which will inform the design of a definitive RCT to evaluate its expected cost effectiveness in the future.

### Continuation criteria

We will use continuation criteria to assess if further evaluation of this intervention is warranted, through a formal RCT. The criteria for continuation will be based around feasibility. We will use quantitative and qualitative process evaluation data to consider the following:

- Successful recruitment and retention of 14 general practices and of patients with poor control of T2DM
- Implementation of the intervention as planned
- Acceptability of the intervention for GPs as per the process evaluation
- Potential effect on patient’s glycaemic control and medication intensification
- Exploratory economic analysis indicates that the intervention could be cost effective

### Ethics and data management

Ethical approval has been granted by the ICGP Research Ethics Committee.

A study steering committee will agree on the final data management plan. Currently, the data received from practices (collected by recruited GPs) will be completely anonymised. A unique study identifier number will be given to each GP by the study team, to record each patient participant (this will be recorded in the data collection tool for baseline data measurement). On follow-up, intervention GPs will use the unique patient ID to input the outcome data. Qualitative evaluation data from intervention GPs will be collected through semi-structured interviews which will be audio recorded and transcribed. All practice and anonymised patient data will be stored on secure password-protected hard drives and transferred to a secure password-protected server. Hard copies of data (i.e. completed questionnaires and practice consent forms) will be scanned and stored electronically on a secure password-protected server in accordance with data protection policies; the original copies will be shredded.

## Discussion

Poorly control T2DM is a major contributor to both morbidity and mortality of patients and increasing economic costs [1]. Few professionally targeted interventions have been developed, which specifically focus on patients with poor control of T2DM [26]. Similarly, there is limited evidence on the effect of interventions, which aim to support the intensification of medications and address clinical inertia in T2DM management. We have reported the findings of a non-randomised feasibility study of a complex intervention, which aims to support GP-based management of poorly controlled T2DM. A web-based CDSS, called the DECIDE intervention, was developed to support evidence-based prescribing for patients with poorly controlled T2DM. The non-randomised feasibility study showed that focussing on the medication intensification in poorly controlled T2DM in general practice is suitable, but should also consider non-pharmacological intensification options, such as more frequent reviews and referral to other healthcare professionals. We have then reported the protocol for a cRCT, which aims to assess the feasibility of a DECIDE study. If the study is feasible and the trial methodologies are robust, we will aim to proceed to a full cluster RCT. With the prevalence of diabetes rising globally to 422 million affected adults in 2014, compared to 108 million in 1980, the need for interventions to address the causes of poor control of diabetes are warranted—especially for a cohort of patients who are especially vulnerable to the effects of micro- and macro-vascular complications of poorly controlled T2DM [1]. DECIDE will also not focus on aggressive reductions in HbA1c on those with moderate control to T2DM, aiming to reduce the potential for harm in borderline populations [24–26].

Limitations of the study include the potential that the use of the finder tool in the control practices could contaminate this group and promote a heightened attention on the identified patients. However, we will ask that the finder tool be run by practice nurses only and none of the educational or CDSS elements (e.g. the DECIDE folder or website training) will be available to control practices so treatment will continue as usual. The patients in the control group will continue to receive usual care, which is a six monthly review in the practice. Control practices will be offered access to the DECIDE intervention following study completion. We plan on performing a process evaluation, which will also identify if contamination occurs. Prescribing is a very complex task—especially for patients with poorly controlled T2DM who may also have complex social needs [6]. Though prescribing options are complex in the CDSS, the feedback from the non-randomised feasibility study was that intensification suggestions were logical and not over-burdensome for the intervention GPs. As the reasons for poor control can be multi-faceted and complex, the requirement to address non-pharmacological intensifications options was identified through the non-randomised feasibility study and these options will be provided to intervention GPs in the cRCT.

In summary, DECIDE aims to support GPs in Irish Primary Care to intensify medications, where appropriate, for patients with poorly controlled T2DM, mediated through a complex intervention comprising a CDSS and an educational component.

## Abbreviations

BP: Blood pressure
CDSS: Clinical decision support system
CONSORT: Consolidated standards for Reporting Trials
DECIDE: ComputeriseD dECisIonal support for poorly controlleD typE 2 Diabetes mellitus in Irish General Practice
EHR: Electronic health record
GP: General practitioner
HRB: Health Research Board
ICC: Intra-cluster coefficient
ICGP: Irish College of General Practitioners
NICE: National Institute for Health and Clinical Excellence
T2DM: Type 2 diabetes mellitus
